# Transcription Profiles Associated with Inducible Adhesion in Candida parapsilosis

**DOI:** 10.1128/mSphere.01071-20

**Published:** 2021-02-10

**Authors:** Joseph M. Bliss, George A. Tollefson, Abigail Cuevas, Sarah J. Longley, Matthew N. Neale, Alper Uzun, Sunil K. Shaw

**Affiliations:** a Department of Pediatrics, Women & Infants Hospital of Rhode Island, Providence, Rhode Island, USA; b Warren Alpert Medical School, Brown University, Providence, Rhode Island, USA; c Center for Computational Molecular Biology, Brown University, Providence, Rhode Island, USA; University of Georgia

**Keywords:** *Candida parapsilosis*, cell adhesion, gene expression

## Abstract

Candida parapsilosis has emerged as a frequent cause of invasive candidiasis with increasing evidence of unique biological features relative to C. albicans. As it adapts to conditions within a mammalian host, rapid changes in gene expression are necessary to facilitate colonization and persistence in this environment. Adhesion of the organism to biological surfaces is a key first step in this process and is the focus of this study. Building on previous observations showing the importance of a member of the *ALS* gene family in C. parapsilosis adhesion, three clinical isolates were cultured under two conditions that mimic the mammalian host and promote adhesion, incubation at 37°C in tissue culture medium 199 or in human plasma. Transcriptional profiles using RNA-seq were obtained in these adhesion-inducing conditions and compared to profiles following growth in yeast media that suppress adhesion to identify gene expression profiles associated with adhesion. Overall gene expression profiles among the three strains were similar in both adhesion-inducing conditions and distinct from adhesion-suppressing conditions. Pairwise analysis among the three growth conditions identified 133 genes that were differentially expressed at a cutoff of ±4-fold, with the most upregulated genes significantly enriched in iron acquisition and transmembrane transport, while the most downregulated genes were enriched in oxidation-reduction processes. Gene family enrichment analysis identified gene families with diverse functions that may have an important role in this important step for colonization and disease.

**IMPORTANCE** Invasive *Candida* infections are frequent complications of the immunocompromised and are associated with substantive morbidity and mortality. Although C. albicans is the best-studied species, emerging infections by non-*albicans Candida* species have led to increased efforts to understand aspects of their pathogenesis that are unique from C. albicans. C. parapsilosis is a frequent cause of invasive infections, particularly among premature infants. Recent efforts have identified important virulence mechanisms that have features distinct from C. albicans. C. parapsilosis can exist outside a host environment and therefore requires rapid modifications when it encounters a mammalian host to prevent its clearance. An important first step in the process is adhesion to host surfaces. This work takes a global, nonbiased approach to investigate broad changes in gene expression that accompany efficient adhesion. As such, biological pathways and individual protein targets are identified that may be amenable to manipulation to reduce colonization and disease from this organism.

## INTRODUCTION

*Candida* spp. are frequently found on human skin and in mucosal microbiota in the absence of apparent disease. When host defenses are weakened, however, yeast can leave their commensal niche and cause invasive infection. *Candida* spp. cause an estimated 50,000 invasive infections and 15,000 deaths at a cost of $2 billion per year in the United States (1). Infections caused by C. albicans continue to dominate in many settings, but non-*albicans Candida* species such as C. parapsilosis, C. glabrata, C. krusei, C. tropicalis, and C. auris are emerging as significant causes of systemic infection, especially in the setting of immune compromise (2). Resistance against first-line and second-line antifungals is increasing in C. parapsilosis and C. glabrata, and multidrug resistance has been reported for C. auris (3, 4). Non-*albicans Candida* species exhibit unique biology and distinct mechanisms of pathogenesis but are poorly understood.

Among the non-*albicans Candida* species, C. parapsilosis has generated interest based on unique aspects of its biology and emergence in many parts of the world as an invasive pathogen nearing or exceeding the incidence of C. albicans (5). It is also a frequent cause of disease in neonatal intensive care units (6). Among its clinically relevant attributes are the ability to form biofilms on central venous catheters and other medical devices and its capacity for horizontal transmission from environmental sources, including soil, water, and the hands of health care workers (7–9). Because C. parapsilosis maintains a reservoir in the environment, rapid modifications in gene expression are likely to be necessary to adapt to niches within a host. Adhesion is a necessary first step in successful colonization and potential invasive disease in a susceptible host, leading our group and others to investigate the mechanisms involved in adhesion of the organism to mucosal and biotic surfaces both *in vitro* and *in vivo* (10–13).

We recently described an adhesion assay to measure binding of C. parapsilosis to extracellular matrix proteins such as albumin and fibronectin under fluid shear (13). We observed that certain clinical isolates of C. parapsilosis exhibited enhanced adhesion when grown in mammalian tissue culture medium 199 (M199) or human serum. The *ALS* gene family is well known to encode important adhesins in C. albicans (14), and recent work has defined the unique features of the *ALS* family in C. parapsilosis (15). We found that *CpALS7* (*CPAR2_404800* or *CpALS4800* [15]) was significantly upregulated when a clinical C. parapsilosis isolate was grown under conditions that led to adhesion (13). Because these conditions model the host environment to which yeast must adapt to successfully cause systemic disease, we hypothesized that clinical isolates of C. parapsilosis differentially regulate many genes that are important for virulence in the presence of these host stimuli. In the current study, we used RNA-seq to compare the transcriptional profiles of three adhesive clinical isolates of C. parapsilosis under adhesion-promoting or adhesion-suppressing growth conditions to identify gene expression patterns associated with these phenotypes.

## RESULTS

### Adhesion of C. parapsilosis isolates.

**FIG 1:**
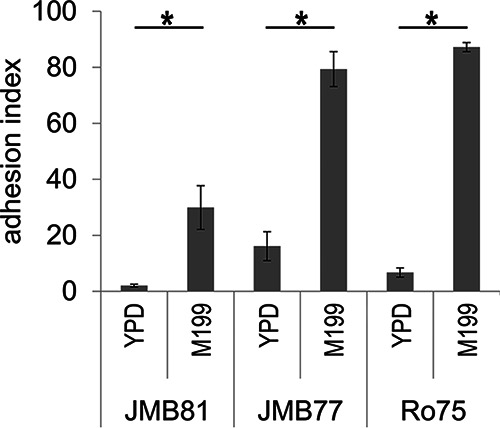
Adhesion under fluid shear of C. parapsilosis clinical isolates. Isolates were grown in YPD or M199 for 3 h, and then adhesion was measured under fluid shear. Adhesion index (*y* axis) represents the percentage of the flow channel surface that was covered with yeast. The graph depicts the means of 4 independent experiments with duplicate channels; error bars represent the standard error of the mean (SEM). Adhesion assays and qPCR validations (Fig. 6) were performed on samples prepared in parallel. Comparisons were made with analysis of variance (ANOVA). Between-group comparisons were made with the Holm-Sidak test. *, *P < *0.001.

To confirm that adhesion was induced in the same samples that were to undergo transcriptional profiling, adhesion of all 3 clinical isolates of C. parapsilosis to immobilized bovine serum albumin (BSA) was measured under physiologic fluid shear as previously reported (13). All three isolates showed relatively weak adhesion when grown in yeast extract-peptone-dextrose (YPD). After 3 h growth in M199, all isolates showed significantly increased adhesion compared to YPD (Fig. 1). JMB81 showed a moderate increase in adhesion in M199, but JMB77 and Ro75 showed much greater increases, with 80 to 90% of the entire channel surface area carpeted with yeast.

### Transcriptional profiling of C. parapsilosis by RNA-seq.

Total RNA was prepared from all three isolates grown in YPD, M199, or pooled human plasma in three independent biological replicates. Next-generation sequencing using the Illumina platform was completed as described in Materials and Methods. Summary characteristics of the RNA-seq data are provided in Table 1.

**TABLE 1:** RNA-seq data summary

Isolate	Medium	Total clean reads (millions)	Avg genome coverage (fold)	Genome alignment rate (%)	Aligned sequence reads (millions)
JMB81	YPD^*a*^	42.6	490	89	37.9
		45.5	524	88	40
		39	449	88	34.3
	M199	41.6	479	89	37.1
		42	483	89	37.2
		41.6	479	88	34.9
	Plasma	49.5	570	89	43.8
		45.3	521	89	40.1
		41.6	479	89	32.8
JMB77	YPD	23.8	274	94	22.5
		24.3	280	95	23
		24.1	277	95	22.8
	M199	24.6	283	95	23.3
		24.2	279	94	22.8
		24.5	282	95	23.4
	Plasma	24.6	283	90	22.1
		24.5	282	94	23.1
		24.4	281	95	23.1
Ro75	YPD	24.6	283	95	23.4
		24	276	94	22.6
		24.5	282	95	23.2
	M199	24.4	281	90	22
		24.2	279	95	22.8
		24.8	285	95	23.6
	Plasma	24.7	284	95	23.5
		24.4	281	95	23.2
		24.4	281	95	23

a Data for each biological replicate of each strain and medium are provided.

**FIG 2:**
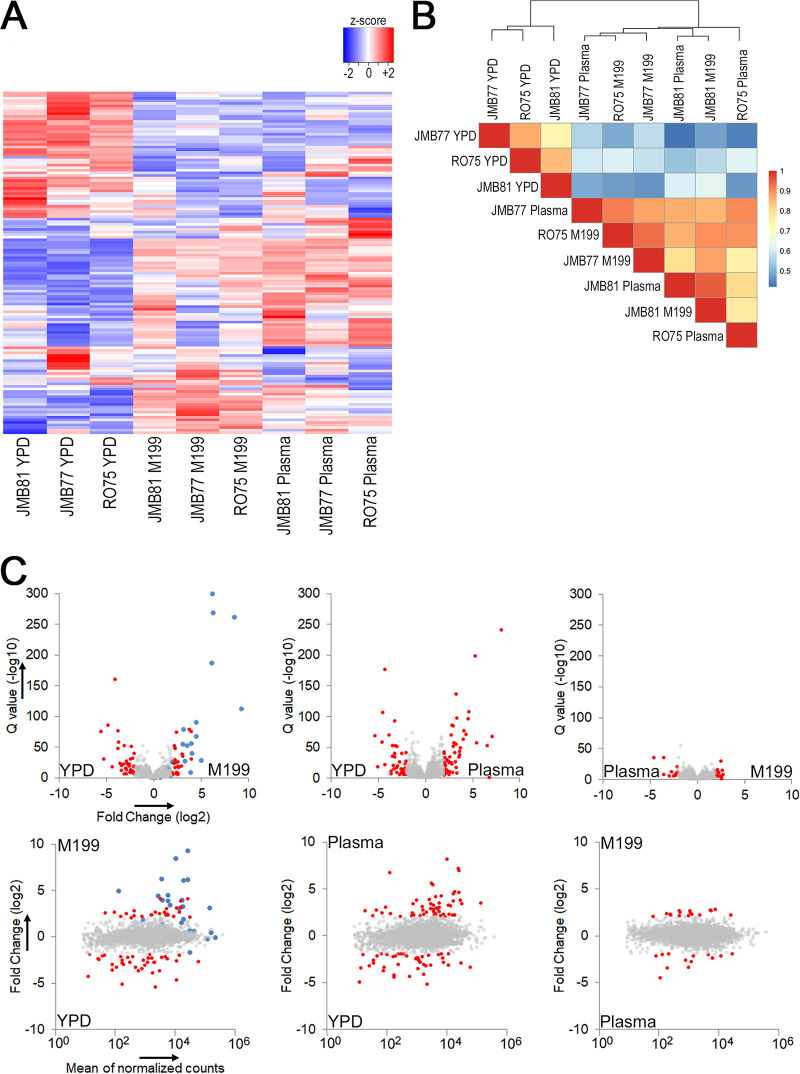
Heat map and correlation profiles of differentially expressed genes. Gene expression profiles of isolates grown under nonadhesive (YPD) or adhesive conditions (M199 and Plasma) were determined using the RNA-seq of three independent replicates. A total of 133 differentially expressed genes were observed between growth conditions. (A) Heat map of the 133 differentially expressed genes, normalized by z scores and ranked by row means. (B) Correlation matrix of the 133 differentially expressed genes to allow visualization of similarities among growth conditions. (C) Volcano plots with gene expression plotted as Q value (upper panels) and MA plots showing fold change (lower panels) comparing all three growth conditions. The red dots indicate genes that had a >±4-fold change in expression. The blue dots represent genes whose expression was validated by qPCR (Fig. 6). The gray dots fall below the ±4-fold threshold.

The gene expression profiles of three adhesive C. parapsilosis isolates were compared in adhesion-inducing or adhesion-noninducing growth conditions. Pairwise differential gene expression analyses of isolates grown in each of the three growth conditions showed that there were 133 differentially expressed genes (log_2_-fold change ±2, adjusted *P* < 0.001) among the three conditions (Table S1). The most upregulated genes in adhesion-inducing conditions were significantly enriched (corrected *P* ≤ 0.05) in iron acquisition and transmembrane transport, while the most downregulated genes were enriched in oxidation-reduction processes. Profiles of differentially expressed genes from isolates grown in adhesion-inducing growth media, M199 and plasma, were similar to each other (Fig. 2A). Their profiles were significantly different from those grown in YPD as illustrated by a correlation plot of the normalized expression counts for the 133 differentially expressed genes (Fig. 2B).

10.1128/mSphere.01071-20.2 TABLE S1 DESeq2 result tables for the 133 differentially expressed genes. The tables include normalized counts for all three replicates of three strains grown in all three conditions as well as differential expression calculations for each pairwise comparison. Annotations containing gene descriptions and orthologue names from the *Candida* Genome Database are included as described in Materials and Methods. The fourth tab contains the sample ID key to identify isolates and conditions for the samples in the DESeq2 result tables. Download Table S1, XLSX file, 0.1 MB. Copyright © 2021 Bliss et al. 2021 Bliss et al. https://creativecommons.org/licenses/by/4.0/ This content is distributed under the terms of the Creative Commons Attribution 4.0 International license.

Volcano and MA plots (Fig. 2C) of the expression data show that the majority of differentially expressed (DE) genes in our comparisons were those differentially expressed between nonadhesive and adhesive growth conditions, YPD versus M199 (78 DE genes) and YPD versus plasma (96 DE genes), while relatively few genes were differentially expressed between the two adhesion-inducing growth media, M199 and plasma (28 DE genes). The MA plots show that many of the genes that were differentially expressed in both of the adhesive versus nonadhesive condition comparisons were among the most highly expressed genes in the genome.

The 20 genes that demonstrated the highest upregulation based on fold increase in expression in adhesive conditions (M199, plasma) compared to nonadhesive conditions (YPD) are listed in Table 2 along with their respective normalized count values from the differential expression analysis R package, DESeq2. They were ranked by a ratio of mean fold change between adhesive and nonadhesive conditions in descending order. Homologues of each gene in C. albicans, and Saccharomyces cerevisiae are indicated where known. The genes that showed the highest upregulation in adhesive conditions included homologues of *INO1*, *PHO89*, *PHR1*, and *CDG1.* Each showed a 50-fold or greater increase in mean normalized count values. Complete tables of differential expression results for each of the three pairwise comparisons are included in the supplemental material (Data Set S1).

**TABLE 2:** Normalized counts of genes showing the greatest upregulation in adhesive conditions^*c*^

	JMB81			JMB77			Ro75			Mean adh	Mean non	Adh/non	Homologues	
Gene^*a*^	YPD	199^*b*^	P	YPD	199	P	YPD	199	P				*C.alb*	*S.cer*
*200650*	151	68,159	13,332	86	76,293	16,535	76	45,841	12,737	38,816	105	371	*INO1*	*INO1*
*400510*	80	27,293	31,662	35	13,396	8,052	28	8,847	5,885	15,856	48	333	*PHO89*	*PHO89*
*302140*	431	37,441	53,724	565	26,366	48,212	277	25,821	42,625	39,031	424	92	*PHR1*	
*800950*	1	52	238	6	170	321	3	87	306	196	3	57		
*701230*	34	2,403	2,097	128	9,483	3,860	119	9,710	4,305	5,310	94	57	*CDG1*	
*300630*	1,728	51,656	26,948	286	34,006	11,066	369	34,007	10,710	28,066	794	35		
*703800*	162	1,267	4,140	147	3,134	8,206	121	3,261	8,886	4,816	143	34		
*402000*	2,240	38,267	48,496	2,983	29,616	46,107	1,003	29,268	45,663	39,569	2,075	19	*PGA30*	
*210080*	281	4,515	1,827	163	5,823	3,753	223	4,340	3,724	3,997	222	18	*ITR1*	*ITR2*
*300120*	605	12,680	28,475	1,824	22,227	26,447	2,087	31,695	26,898	24,737	1,505	16	*CSA1*	
*600450*	954	7,376	15,842	730	5,986	13,692	458	8,834	8,265	9,999	714	14	*HGT10*	*STL1*
*500330*	579	10,251	3,321	588	16,335	1,728	738	14,997	2,009	8,107	635	13		
*106700*	357	8,728	6,719	627	6,150	3,248	278	5,142	2,460	5,408	421	13		
*105760*	1,995	34,405	46,803	3,276	13,791	21,822	1,266	16,753	17,302	25,146	2,179	12	*ENA2*	*ENA2*
*701390*	354	1,884	4,454	1,026	4,157	13,306	357	4,044	11,568	6,569	579	11		
*402910*	21,082	171,554	299,494	18,354	167,371	201,154	25,263	20,9671	183,140	205,397	21,566	10		
*300130*	1,449	18,524	25,790	1,444	10,902	12,470	2,664	17,938	13,360	16,497	1,853	9	*FRP2*	
*402900*	2,258	20,281	29,323	1,976	16,217	15,862	2,550	19,979	14,639	19,383	2,261	9	*PGA7*	
*602990*	4,374	26,849	15,162	1,887	35,448	10,240	1,968	42,436	9,699	23,306	2,743	8	*CTR1*	*CTR1*
*108340*	220	5,638	4,269	2,524	8,558	8,768	295	13,577	8,289	8,183	1,013	8		

a Gene numbers each follow “Cpar2_” nomenclature (45).

b Shaded rows show adhesive conditions.

c Abbreviations: YPD, yeast peptone dextrose; 199, medium 199; P, plasma; adh, adhesive conditions; non, nonadhesive conditions (YPD); *C.alb*, C. albicans; *S.cer*, S. cerevisiae.

10.1128/mSphere.01071-20.1 DATA SET S1 Complete DESeq2 result tables. The tables include normalized counts for all three replicates of three strains grown in all three conditions as well as differential expression calculations for each pairwise comparison. Annotations containing gene descriptions and orthologue names from the *Candida* Genome Database are included as described in Materials and Methods. The fourth tab contains the sample ID key to identify isolates and conditions for the samples in the DESeq2 result tables. Download Data Set S1, XLSX file, 10.0 MB. Copyright © 2021 Bliss et al. 2021 Bliss et al. https://creativecommons.org/licenses/by/4.0/ This content is distributed under the terms of the Creative Commons Attribution 4.0 International license.

**FIG 3:**
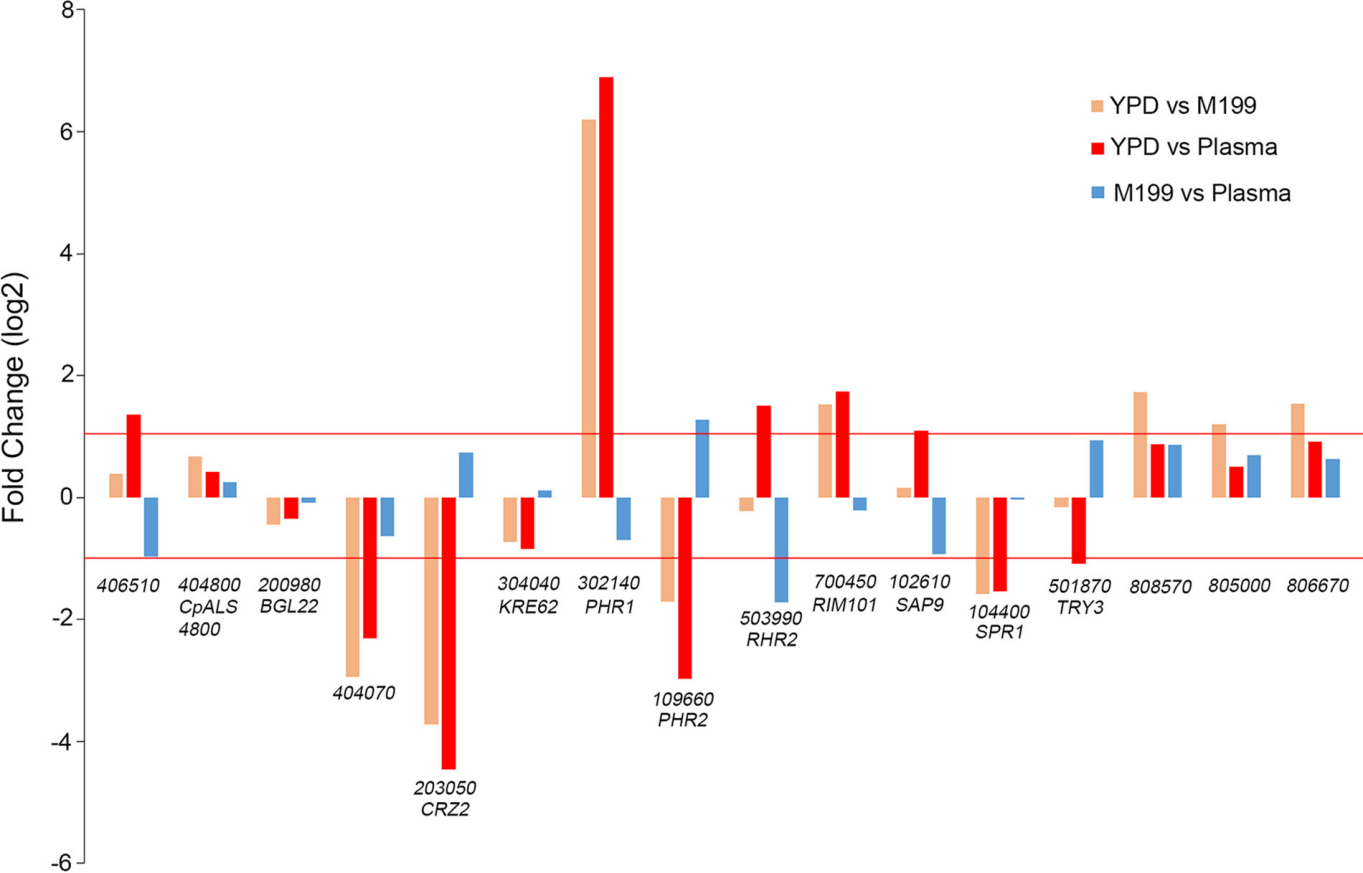
Adhesin gene expression fold change in three condition comparisons. A total of 16 genes with suggested involvement in adhesion based on the *Candida* Genome Database were selected from RNA-seq data, and gene expression log_2_-fold change values were plotted for each gene in each of the following three condition comparisons: YPD versus M199, YPD versus plasma, and plasma versus M199. Red lines mark a ±2-fold gene expression change threshold. The *x* axis labels reflect the *CPAR2* gene designation and the corresponding homologous gene name, where known.

Genes that have been previously suggested to have a role in adhesion of *Candida* species were identified using Gene Ontology annotations from the *Candida* Genome Database. The expression of the homologous genes in C. parapsilosis was compared in YPD, M199, and plasma in this subset of genes (Fig. 3). As expected, a number of these genes showed a greater than 2-fold change in expression in M199 or plasma compared to YPD. Very few genes reached this threshold when expression in M199 and plasma was compared.

**FIG 4:**
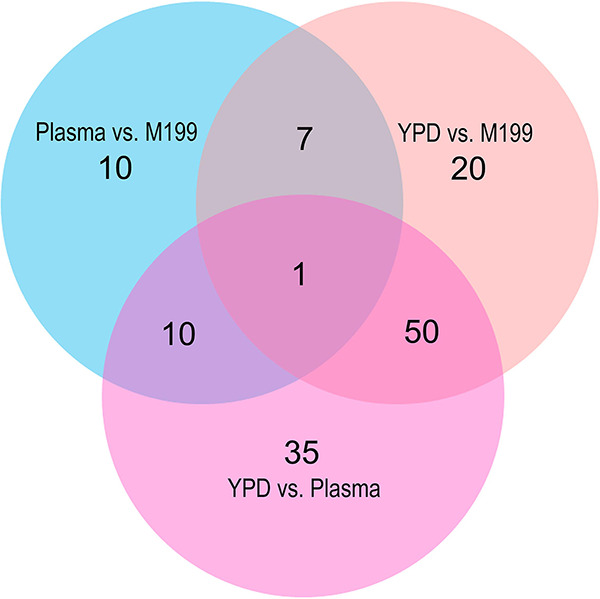
Venn diagram of differentially expressed genes. The distribution of individual genes among the different growth conditions is depicted.

A comparative analysis of the results of each of the three differential expression comparisons using a three-set Venn diagram (Fig. 4) showed that, of the 105 genes which were differentially expressed in isolates grown in adhesive growth conditions compared to those grown in nonadhesive growth conditions, 50 genes were differentially expressed in both YPD versus M199 and in YPD versus plasma. There were 35 unique genes which were differentially expressed in YPD versus plasma and 20 unique genes which were differentially expressed in YPD versus M199.

The most enriched GO terms among each of the differential expression comparisons for each set of differentially expressed genes are included in the supplemental material (Table S2).

10.1128/mSphere.01071-20.3 TABLE S2 Gene Ontology (GO) term enrichment analysis result tables. The table depicts GO terms for significantly up- or downregulated genes for each of the three pairwise condition DESeq2 comparisons. Download Table S2, XLSX file, 0.1 MB. Copyright © 2021 Bliss et al. 2021 Bliss et al. https://creativecommons.org/licenses/by/4.0/ This content is distributed under the terms of the Creative Commons Attribution 4.0 International license.

### Gene family enrichment analysis.

**FIG 5:**
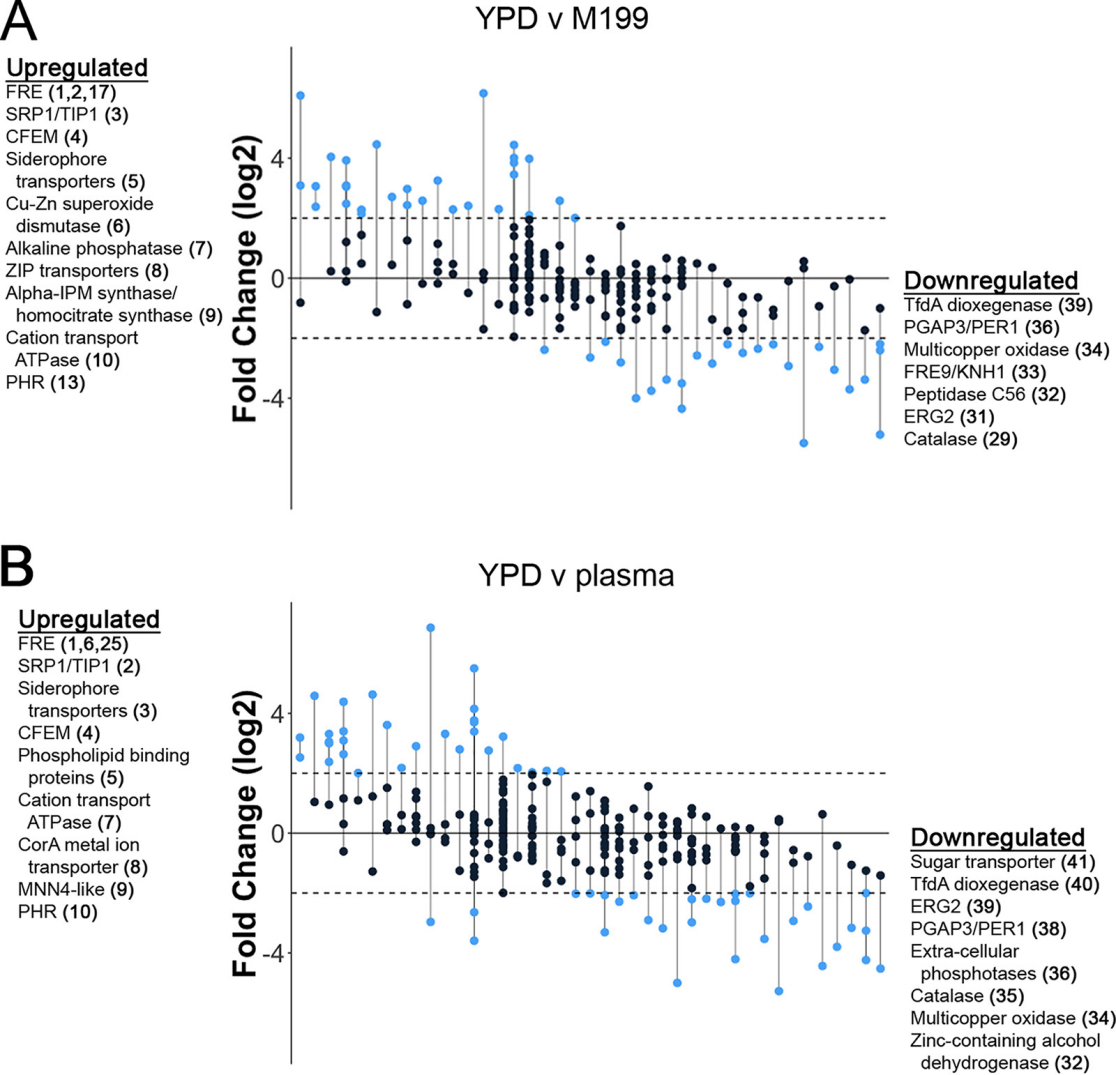
Gene family enrichment analysis of differentially expressed genes. Gene families of differentially expressed genes between two nonadhesive and adhesive growth condition comparisons are depicted. (A) YPD versus M199; (B) YPD versus plasma. Gene families are ordered from left to right in descending order according to their average fold change. Each vertical line represents a gene family or subfamily. Circles represent individual genes within each family. Blue circles represent genes that were differentially expressed by at least ±4-fold. The most upregulated and downregulated families are listed on each side of the plots. The sequential order on the *x* axis, in which the gene families appear from left to right, is listed in parentheses next to each gene family label.

Since C. albicans orthologue annotations are typically used to identify gene family members, genes that are unannotated in C. albicans or unique to C. parapsilosis may be overlooked which exist in biologically significant gene families. Gene families of differentially expressed genes in C. parapsilosis for each of the three condition comparisons were identified using BLASTp similarity searches and the Markov clustering algorithm (16–18) as described in Materials and Methods. For each of the three comparisons, gene families of differentially expressed genes were arranged from highest to lowest change in expression (Fig. 5). The FRE, CFEM, PHR, siderophore transporters, and MNN4 families were among the most highly upregulated gene families in adhesion-inducing conditions. Detailed gene family clustering results are included in the supplemental material (Table S3).

10.1128/mSphere.01071-20.4 TABLE S3 Gene family clustering result tables. The table depicts genes found to be grouped in gene family clusters by homology for each condition comparison as described in Materials and Methods. The gene groups are labeled with known gene family names. The data tables also contain fold change and mean expression values from DESeq2 for each gene as well as orthologue gene names and gene descriptions from the *Candida* Genome Database. Individual genes within gene families that were differentially expressed in our analysis are highlighted in yellow. Download Table S3, XLSX file, 0.1 MB. Copyright © 2021 Bliss et al. 2021 Bliss et al. https://creativecommons.org/licenses/by/4.0/ This content is distributed under the terms of the Creative Commons Attribution 4.0 International license.

### Validation by qPCR.

**FIG 6:**
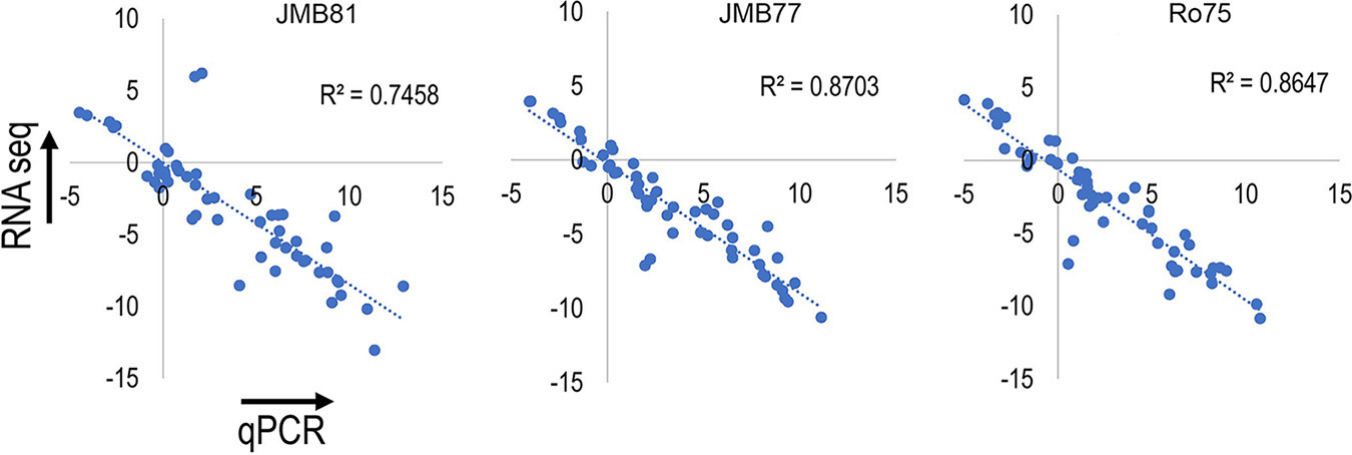
qPCR validation of RNA-seq data. A total of 26 genes were selected from RNA-seq data, and transcript levels were measured using qPCR on 3 independent biological replicates grown in YPD or M199. Strong linear correlation as measured by R^2^ values was observed between qPCR and RNA-seq. qPCR units are displayed as Δ*C_t_* relative to actin control, and RNA-seq expression levels, as FPKM-actin (Log_2_ transformed).

To provide independent validation of the RNA-seq data, the expression of 26 genes that showed various degrees of changes in expression was measured by qPCR specific for each gene. Figure 6 shows correlation plots comparing qPCR (Δ*C_t_* relative to actin) to RNA-seq data (fragments per kilobase per million [FPKM] values for each gene minus actin). In each isolate, the correlation between qPCR and RNA-seq was high, with R^2^ values greater than 0.74 for all isolates.

### qPCR of select differentially expressed genes in a nonadhesive strain.

**FIG 7:**
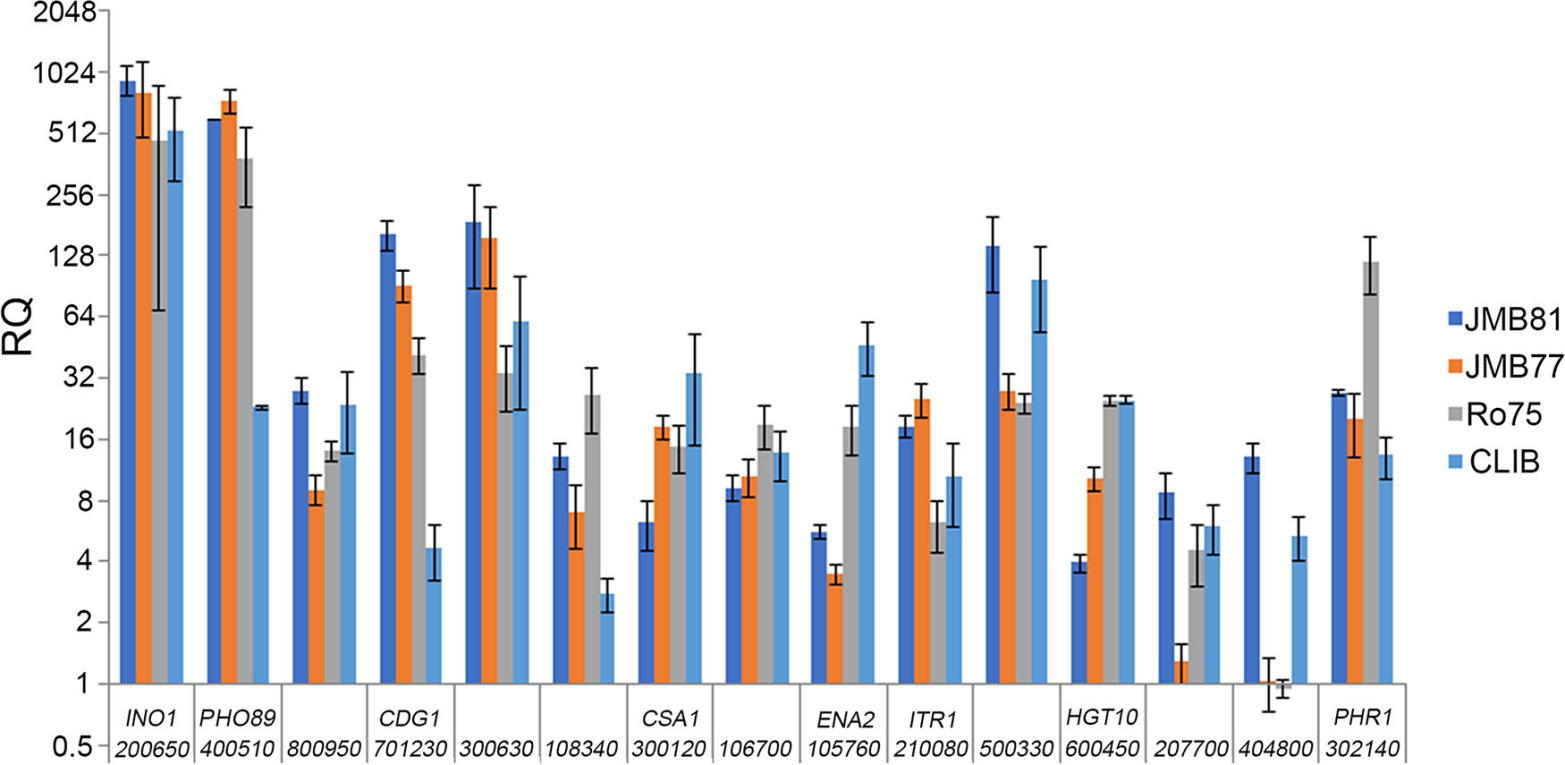
qPCR of upregulated genes on nonadhesive strain. A total of 15 genes were selected from the RNA-seq data that were strongly upregulated in the adhesive strains grown in M199, and transcript levels were measured using qPCR on 3 independent biological replicates grown in YPD or M199. The expression of each gene was compared to the expression in the nonadhesive strain, CLIB214 (CLIB). Relative quantification (RQ) is shown on a log_2_ scale. The *x* axis labels reflect the *CPAR2* gene designation and the corresponding homologous gene name, where known.

Genes that are differentially expressed in adhesion-inducing conditions are strong candidates to have a role in adhesion. Identifying genes that are strongly upregulated in adhesive strains but to a lesser extent in strains that do not have an inducible adhesive phenotype is one method to further narrow the list of candidate genes. To explore the potential utility of this approach, qPCR was performed to measure the relative expression of a subset of the strongly upregulated genes identified in the adhesive strains compared to a strain that demonstrates only weak inducible adhesion, CLIB214 (Fig. 7). Several genes (*Cpar2_400510*, *Cpar2_701230*, *Cpar2_108340*) showed considerably higher upregulation in all three adhesive strains than in CLIB214. Analyses such as these will be helpful in prioritizing additional mechanistic studies.

### Comparison of transcriptional responses of C. parapsilosis and C. albicans.

The C. albicans transcriptome was evaluated previously in response to a variety of conditions known to trigger filamentation compared to yeast growth in YPD alone at 30°C for 3 h (19). While the growth conditions used in that experiment were not identical to those in our study, their growth conditions were the most similar to ours among the data sets which were made available in public repositories. To allow a first approximation of similarities and differences in the response to these stimuli between species, the differentially expressed genes for C. albicans grown in liquid RPMI or YPD containing 10% fetal bovine serum at 37°C were compared to the differentially expressed genes identified in the current study for C. parapsilosis grown in M199 or human plasma. Genes that were coordinately up- or downregulated in both species as well as genes that were divergently regulated are shown in Table 3.

**TABLE 3:** Comparison of transcriptional responses of C. parapsilosis to C. albicans

Similar				Divergent			
Increased		Decreased		*Cp* ↑/*Ca* ↓		*Ca* ↑/*Cp* ↓	
Gene^*a*^	Function	Gene	Function	Gene	Function	Gene	Function
*PHO89*	Phosphate permease	*FRE10*	Ferric reductase	*HGT10*	Glycerol permease	*HWP1*	Hyphal cell wall protein
*CDG1*	Cysteine dioxygenase	*CRZ2*	Transcription factor	*CDG1*	Cysteine dioxygenases	*RBT5*	Member of CFEM family
*PHR1*	Cell surface glycosidase	*LDG3*		*LYS22*	Homocitrate synthase	*ATO1*	Transmembrane protein
*800950*				*CUP9*	Transcription factor, suppresses filamentation	*208810*	
*PGA30*	GPI-anchored protein					*HSP70*	hsp70 chaperone
*ENA2*	Sodium transporter					*PGA34*	GPI-anchored protein
*FRP2*	Ferric reductase					*MNN1*	Alpha-1,3-mannosyltransferase
*FRP1*	Ferric reductase					*GUD1*	Guanine deaminase
*PGA7*	CFEM family member					*PGA23*	GPI-anchored protein of unknown function
*MRV2*						*FAV2*	Adhesin-like protein
*SIT1*	Ferrichrome siderophore transporter					*202920*	
*600590*	Hap43p-induced gene					*GLX3*	Glyoxalase III activity, chaperone activity
*UME6*	Transcription factor, promotes filamentation					*HGC1*	Hypha-specific G1 cyclin-related protein

a Gene names are provided where available; otherwise, the Cpar2_ numeric designation is listed (45).

## DISCUSSION

Previous studies have examined the C. parapsilosis transcriptome. These reports have defined responses to different growth media, temperatures, hypoxia (20, 21), quorum sensing molecules (22, 23), and changes associated with acquired resistance to azole drugs (24). Indeed, these studies have contributed substantially to detailed annotation of the C. parapsilosis genome, and the effects of mutations in key elements of virulence pathways have been globally defined. Several studies have focused on specific transcription factors and their targets (21, 25–29). An additional study characterized the response to a monocyte cell line *in vitro* to identify potential virulence mechanisms (30). While these reports have provided important insights into the gene regulation and pathobiology of C. parapsilosis, the majority have focused on one or two strains (CLIB214 or CDC317). However, a study comparing genomic sequence data from 3 additional isolates that varied in source and geographic region demonstrated substantial genomic variability, including variations in copy number, rearrangements, and single nucleotide polymorphisms (31). The phenotypic variability in adhesion observed in the current report in clinical isolates is striking and may reflect genomic or gene expression variability that has not been previously detected. Additionally, in many studies of the C. parapsilosis transcriptome mentioned above, yeasts were grown in standard laboratory medium and at 30°C. We have focused our studies on conditions that are likely to more closely mimic a host environment, including growth in mammalian tissue culture medium or human plasma and at physiologic temperatures of 37°C. These environmental cues are likely to reveal additional gene expression changes that are relevant to virulence.

In the current study, to model the early transcriptional response within the host, we used a strategy of examining adhesive clinical isolates, maintained at low passage number and grown under conditions modeling the host environment. We previously reported that growth in M199 or fetal bovine serum (FBS) at 37°C resulted in enhanced adhesion to immobilized substrates under fluid shear compared to growth in YPD (13). In the current study, human plasma was substituted for FBS to more closely mimic human conditions and include any potential influence of plasma components that would be lost during serum preparation (32). Although an effect similar to that of FBS is likely, we were unable to experimentally confirm the adhesion phenotype *in vitro* because the microfluidics adhesion assay is sensitive to shear forces that were significantly altered due to the increased viscosity of plasma. Comparison of the transcriptome under these conditions allowed identification of genes that were differentially expressed during the switch to an adhesive phenotype. In each of the growth conditions tested (YPD, M199, or plasma), common sets of genes were differentially expressed in all three strains, although some variations were also observed among them. Interestingly, for all three strains, transcriptional profiles in M199 and plasma resembled one another more closely than those in YPD. In combination with our earlier observation of increased adhesion (13), this result indicates that some of the differentially expressed genes in M199 and plasma are likely to represent adhesins or adhesion-related factors, although many others appear to encode transporters and genes involved with nutrient acquisition.

The *ALS* gene family is well known for its role in adhesion in C. albicans (33). Recent work using a combination of approaches to avoid pitfalls associated with accurate assembly of genome sequence data for these genes having multiple similar loci and tandem copies of repeated sequences has defined 5 *ALS* genes in several C. parapsilosis strains (15). Because these genes were substantively different from the C. albicans *ALS* genes, a naming scheme was proposed to capture their genomic arrangement (*CpALS4770*, *CpALS4780*, *CpALS4790*, *CpALS4800*, and *CpALS660*) (15). Careful assessments of C. parapsilosis *ALS* gene expression in this study demonstrated considerable variation among strains, which is much in line with our findings. Consistent with our previous report (13), *CpALS4800* (*CPAR2_404800* or *CpALS7*) was upregulated in JMB81 in M199 and plasma. However, in JMB77 and Ro75, *CpALS4800* expression was consistently high under all conditions despite phenotypic differences in adhesion. This result suggests either that *CpALS4800* does not contribute to adhesion under the conditions of our assay in these strains or that its adhesive function is being modulated by another factor. The other *ALS* family members did not show differential expression in any isolate and were generally expressed at low levels. *CpALS4790* is notable in that it was the most abundant constitutively expressed *ALS* gene in JMB81 (13) and in the strains studied by Oh et al. (15), leading the authors to suggest an abundance of CpAls4790 on the cell surface. Further, a *CpALS4790* null mutant showed a loss of adhesion to epithelial cells *in vitro* and a reduction in fungal burden in a mouse model of vaginal candidiasis (10). However, no *CpALS4790* expression was detected by RNA-seq from JMB77 or Ro75, although adhesion is robust in these clinical isolates, and they were capable of colonization and/or invasive disease in the human infants from whom they were obtained. This finding is also consistent with our previous study, in which genomic sequences for *CpALS4790* were not detected in JMB77 and Ro75 (13). Taken together, these findings lend further support to the heterogeneity of the *ALS* family among isolates of C. parapsilosis and represent additional evidence to support the noted genomic variability among strains of this species, including the *CpALS* genes (31). The observations also underscore the complexity of the adhesive phenotype and the difficulty in attributing it to a single gene product or family.

Several members of the *CFEM* (common in fungal extracellular membranes) gene family were strongly upregulated in adhesive conditions—*CFEM2*, *CFEM3*, and *CFEM6* (*CPAR2_402910*, *CPAR2_402900*, and *CPAR2_300120*, respectively). These genes are syntenic with the C. albicans genes *RBT5*, *PGA10*, and *CSA1* and are involved in heme binding and iron acquisition, a role that is conserved between the species. These genes have been shown to be upregulated in response to *BCR1*, a transcription factor that regulates biofilm formation (25). Iron acquisition from the host is increasingly being recognized for its importance in virulence among pathogenic fungi and is integrated with alterations in the cell surface related to morphogenesis and adhesion (34). Further, it has been shown that iron modulates chromatin activation of known adhesins and putative adhesin-like cell surface proteins in C. albicans (35). The finding that genes involved in iron acquisition were so strongly upregulated in our experimental conditions supports the notion that these conditions mimic the host environment and are useful for identification of virulence pathways.

The *PHR* family of transglycosylases is important in the virulence of C. albicans and is necessary for processing of β-1,3-glucans in the yeast cell wall (36). In the current study, *CpPHR1* (*CPAR2_302140*) and *CpPHR2* (*CPAR2_109660*) showed reciprocal transcriptional regulation. The C. albicans *PHR* genes exhibit inverse pH-dependent expression (36), and our results suggest that they behave similarly in C. parapsilosis. A *PHR1* null mutant in C. parapsilosis forms biofilms with reduced numbers of cells, implying a role in this function. (37) Another glucan-processing enzyme that was upregulated under adhesive conditions was *CpSKN1* (*CPAR2_807200*). *SKN1* serves as a β-1,6-glucan synthase in C. albicans and functions in concert with the constitutive β-1,6-glucan synthase, *KRE6.* (38) Consistent with our findings, expression of *SKN1* in C. albicans is induced upon induction of hyphal growth. The current study supports the possibility that polysaccharide remodeling of the cell wall, which is necessary for hyphal growth in C. albicans, may be important in adhesion of C. parapsilosis.

Other groups have examined the C. albicans transcriptional response, under similar but not identical conditions. Azadmanesh et al. compared the profile of SC5314 grown in YPD at 30°C for 3 h to growth in RPMI or in YPD containing 10% FBS at 37°C for 3 h (19). Despite these differences, our data show similar responses between C. albicans and C. parapsilosis for many of the top differentially expressed genes, including *PHO89*, *CDG1*, and *PHR1* (Table 3). These results raise the possibility of a generic *Candida* response to host stimuli. However, several genes show a disparate response between the two species. *CUP9* encodes a transcription factor that was upregulated in C. parapsilosis under adhesive conditions but is downregulated in C. albicans, where it represses filamentation and expression of hypha-specific genes such as *HWP1* and *ECE1*. (39) Genes encoding two cell wall proteins, *HWP1* and *RBT5*, are both strongly upregulated in C. albicans but show little change in C. parapsilosis, perhaps an effect of the upregulation of *CpCUP9*. Thus, these results support both general *Candida* spp. responses and C. parapsilosis-specific responses.

In summary, we describe a new approach to identifying C. parapsilosis adhesins, utilizing clinical isolates that show strong adhesion *in vitro* when exposed to host factors. Using this approach, several potential adhesins and transcription factors were identified that may play a role in adhesion. It is likely that additional adhesion pathways or host stimulus responses may dominate in different isolates. It will also be useful to compare nonadhesive isolates to identify genes that are not relevant to adhesion.

## MATERIALS AND METHODS

### Strains and growth conditions.

The C. parapsilosis clinical isolates used in the current study (JMB81, JMB77, and Ro75) have been previously described, and CLIB214 is a type strain (13). Isolates were confirmed as C. parapsilosis *sensu stricto* using PCR probes specific to the *ITS1* region (40). Yeasts were grown overnight in YPD medium (1% yeast extract, 2% peptone, 2% dextrose) with vigorous agitation at 37°C. Stationary-phase cultures were washed with sterile water and resuspended in fresh YPD medium, tissue culture medium M199 (BioWhittaker/Lonza, no. BW12-117Q), or pooled human blood plasma (sodium heparin anticoagulated) at 3 × 10^6^/ml and further incubated at 37°C for 3 h without shaking. Under these growth conditions, filaments (hyphae or pseudohyphae) were not observed. Parallel samples were tested in adhesion assays (Fig. 1) to confirm the adhesive phenotype, or yeast were centrifuged and pellets were flash frozen in liquid N_2_ and stored at −80°C for later RNA extraction for RNA-seq or quantitative PCR (qPCR) validation.

### Adhesion assays.

A microfluidics multichannel adhesion assay was used to measure adhesion of yeast under fluid shear, using the Bioflux 200 platform (Fluxion Biosciences) as previously described (13). Briefly, channels were coated with BSA for 48 h and then washed with Hanks’ balanced salt solution with calcium and magnesium (HBSS+) at 2 dynes/cm^2^ to remove unbound protein. Yeasts grown in YPD or M199 were loaded into the channels and passed through at 5 dynes/cm^2^ for 30 min. Excess yeasts were then removed from inlets and outlets, and Dulbecco’s phosphate-buffered saline with calcium and magnesium (DPBS+) containing 5 μM calcofluor white was added to the channels. The flow was resumed for another 10 min at 5 dynes/cm^2^ to remove unbound yeast. The channels were then imaged using a Nikon Ti-E inverted fluorescence microscope with a motorized stage to automate sequential imaging of the channel area. Yeast binding was measured in the DAPI (4′,6-diamidino-2-phenylindole) (calcofluor) fluorescence channel (395 nM excitation, 440/40 emission). An intensity threshold was used to determine the percentage of the channel surface area that was covered with yeast (expressed as adhesion index in Fig. 1). Data from 4 independent experiments with duplicate channels for each condition were pooled. Statistical analysis was conducted using SigmaPlot v13.0.

### RNA extraction, library preparation, and sequencing.

The three strains used in this study were cultured in YPD, M199, or plasma as described above in three biological replicates. Total RNA was extracted from each and purified using a RiboPure yeast extraction kit (Ambion/Life Technologies). RNA integrity was assessed using agarose gels and an Agilent bioanalyzer. RNA-seq library preparation was conducted using the NEBNext Ultra RNA library prep kit for Illumina (New England Biolabs, Ipswich, MA, USA) according to the manufacturer’s instructions, and sequencing was performed following rRNA depletion using paired-end reads (Illumina HiSeq2000), 2 × 150 bp, from all samples.

### RNA-seq pipeline.

Samples were checked for quality control using FastQC v0.11.5 (http://www.bioinformatics.babraham.ac.uk/projects/fastqc/). Samples that contained remaining adaptor sequence were trimmed with Skewer v0.2.1. (41) Reads were then aligned to the genome (20) and quantified using Salmon v1.0.0 (42) in mapping-based mode. Salmon transcript quantification was performed by applying the –gc-bias and –seq-bias flags for correcting fragment-level GC content bias and sequence-specific random hexamer priming biases, respectively. The tximport v1.6.0 R package (43) was used to import transcript quantification files and bias correction matrices into the differential expression analysis workflow. Differential expression analysis using the DESeq2 v1.18.1 R package (44) was performed to identify differentially expressed genes in a series of pairwise comparisons between each of the sets of replicates of three samples in each of the three growth conditions. Default parameters for DESeq2 were used for differential expression analysis.

### Data analysis and annotation.

Gene ontology enrichment was performed using the *Candida* Genome Database’s GO Term Finder tool. (45) Genes which were significantly upregulated or downregulated under each condition comparison were used as a query set for GO term enrichment. GO terms with a Bonferroni corrected *P* ≤ 0.05 were considered to be significantly enriched. C. albicans and S. cerevisiae gene names which are included in the results as annotations were obtained from the *Candida* Gene Order Browser (46, 47).

The gplots v3.0.1 and DESeq2 v1.18.1 R packages were used to create heat maps of the expression data. All R analyses were run using R v3.4.3. To create the heat maps of top differentially expressed genes, variance stabilizing transformation (VST) was first applied to counts of differentially expressed genes which were normalized by DESeq. Then, z scores per expression value were calculated using row means across all samples for each gene and z scores were plotted using the heatmap.2 function contained in the gplots package. The heat map data were clustered using default heatmap.2 settings, which use Euclidean measures to obtain a distance matrix and the complete agglomeration method for clustering, before scaling the data. Finally, the data dendrograms were reordered based on row and column means (https://stat.ethz.ch/R-manual/R-patched/library/stats/html/reorder.dendrogram.html).

Data for the Venn diagram (Fig. 4) were generated through a series of comparisons of the numbers of differentially expressed genes in each of the three DESeq2 results sets. Thresholds of gene expression fold change ≥ ±4 and *P* ≤ 0.001 were used to select genes to include in this comparison. The distribution of shared and unique genes among DESeq2 results sets was displayed as a Venn diagram using the VennDiagram v1.6.20 R package. Additionally, the correlation coefficients of all VST transformed normalized gene counts for each sample were plotted as a clustered heat map using the cor() function of the base R stats library to generate a correlation matrix model and the pheatmap v1.0.12 library to plot a clustered heat map of the correlation coefficient between all samples.

Gene family enrichment analysis of differentially expressed genes was performed by including genes with an expression fold change of ≥±4 and *P* ≤ 0.001 from each of our three condition comparisons in our gene family enrichment analysis workflow. First, same-species homologous protein sequences were determined using BLASTp for the three sets of differentially expressed genes from each DESeq2 comparison (18). Next, significantly enriched gene groups were identified using the Markov clustering algorithm with an inflation factor of 2.1 (16, 17). Finally, the newly identified gene clusters were annotated with known gene family labels using the UniProt database of proteins (48). In some cases, gene family labels did not exist for C. parapsilosis genes, so gene family annotations for homologues in C. albicans or S. cerevisiae were used to identify our gene groups instead. The log_2_-fold change was plotted for genes within significantly enriched gene families using the ggplot2 v3.0.1 R package, and the gene families were ordered from highest to lowest mean log_2_-fold change for each gene group. The gene groups were spaced evenly apart by an arbitrary increment on the *x* axis for enhanced visibility.

### qPCR.

Quantitative PCR was performed as previously described (13). Primer sequences are listed in supplemental material (Table S4).

10.1128/mSphere.01071-20.5 TABLE S4 Primer sequences for qPCR. Download Table S4, DOCX file, 0.1 MB. Copyright © 2021 Bliss et al. 2021 Bliss et al. https://creativecommons.org/licenses/by/4.0/ This content is distributed under the terms of the Creative Commons Attribution 4.0 International license.

### Data availability.

The raw gene expression count files generated from our RNA-seq data are freely available and hosted in the Gene Expression Omnibus (GEO) RNA-seq data repository (accession number GSE159274).
