# Supplementary material for: Transcription Profiles Associated with Inducible Adhesion in Candida parapsilosis
Source: mSphere. 2021 Feb 10;6(1):e01071-20. doi: 10.1128/mSphere.01071-20 (PMC8544891; doi:10.1128/mSphere.01071-20)
Supplement: TABLE S4 [file msphere.01071-20-st004.docx]

Table S4. Primer sequences for qPCR

| **Gene** | **Alternate gene name** | **Name** | **Sequence** |
| --- | --- | --- | --- |
| *CPAR2_200650* | *INO1* | AC1 F | TGGGTGATTCCAAAGTTGCC |
|  |  | AC1 R | TCTTGATCAGACTCGCTCAGT |
| *CPAR2_400510* | *PHO89* | AC2 F | GTGCTGCTGTTACCACTGTT |
|  |  | AC2 R | AAACCAGCCATTGGCAAAGT |
| *CPAR2_800950* |  | AC3 F | AGCTGCATTCACCACTACTT |
|  |  | AC3 R | CCAGCAGCTAAAGCACCAAT |
| *CPAR2_701230* | *CDG1* | AC4 F | TCGAGTGCCATTCATGACCA |
|  |  | AC4 R | ACCAACTTCGTCACGGTACA |
| *CPAR2_300630* |  | AC5 F | GCCGTGCATTGGATTTGGAT |
|  |  | AC5 R | GCAACTCCCACGTACTGATG |
| *CPAR2_108340* |  | AC6 F | GTGTTGGACAAGCTCCCAAA |
|  |  | AC6 R | CAACGCCATAGACGCTGTTA |
| *CPAR2_300120* | *CSA1* | AC7 F | AACTGCTGAAGAGTCGTCCA |
|  |  | AC7 R | TGGCTGAGCACATTCTGGTA |
| *CPAR2_106700* |  | AC8 F | ATGGCCGGAGAATTTACCGA |
|  |  | AC8 R | ATTTATGGCCATCAGCTTGG |
| *CPAR2_105760* | *ENA2* | AC9 F | CCCTTGTTATTGCCCGTTGT |
|  |  | AC9 R | CAGAAGCATCCTTGGCAACA |
| *CPAR2_210080* | *ITR1* | AC10 F | ACAGGTGGTCAATTGGTTGC |
|  |  | AC10 R | TGCCTTGGGTATGTCACCAT |
| *CPAR2_500330* |  | AC11 F | CAAACGTTAGCGGAGTCGTT |
|  |  | AC11 R | TTGATCATCTGGCCCACCAT |
| *CPAR2_600450* | *HGT10* | AC12 F | TTGAAGGTGCTGTGATTGCC |
|  |  | AC12 R | GCAATCAACCAACGAGGTGA |
| *CPAR2_207700* |  | AC13 F | GCACAATTGGCTACGACCAT |
|  |  | AC13 R | GTCAACATTCCCGATTGCGT |
| *CPAR2_403510* | *RBT1* | PK1 F | AAAGCAGACGCCAATTCCAG |
|  |  | PK1 R | CGAAGCTTCACCTTCACCAG |
| *CPAR2_603340* | *PGA59* | PK2 F | ATTGCCACCACCGTTGTTAC |
|  |  | PK2 R | AGCAGAAGTTTCTGGAGCCT |
| *CPAR2_302990* | *PGA56* | PK3 F | CGCTTCATTGGCTTTAGTTG |
|  |  | PK3 R | TTTTTGACCACCAGCACCAC |
| *CPAR2_403180* | *PGA62* | PK4 F | GAACAACCAACCACCGAACA |
|  |  | PK4 R | GCAGCACCCAAAGCCAATAA |
| *CPAR2_302620* | *PGA14* | PK5 F | AGCTCCAACTGTCACTGTCT |
|  | *YNL190W* | PK5 R | CAGCGGTGCTGTTATCTCTC |
| *CPAR2_805040* |  | PK6 F | TGGCTTGTCCAGAATCTGCT |
|  |  | PK6 R | GAACGCTCAATTGCACCAAC |
| *CPAR2_402900* | *PGA7* | PK7 F | CACAATGGGCAGGATTGGTC |
|  |  | PK7 R | TCCTGAAGCAGCAGAAGACA |
| *CPAR2_402910* |  | PK8 F | AATTTGCTGGTGCCATTGGT |
|  |  | PK8 R | GGGTAGTTTCAGCAGCTTCG |
| *CPAR2_107500* | *PGA26* | PK10 F | GTTGCCAGCTTGTCTGCTTA |
|  |  | PK10 R | GGTGACAGTCTTTGCTTCTG |
| *CPAR2_807200* | *SKN1* | PK11 F | ACGAGGGAGGAGCGATAATG |
|  |  | PK11 R | CGAGGAAGCATAGGCGATTG |
| *CPAR2_806670* | *YWP1* | PK12 F | GGAACAGTTGCTGGTGAAGG |
|  |  | PK12 R | AACGGCAGCAGTTGAAGTTT |
| *CPAR2_404800* | *CpALS4800* | ALS7 F | ACCACCACCGAGGTTACAAA |
|  |  | ALS7 R | TGGATCTCCGGTTTTGAGTC |
| *CPAR2_302140* | *PHR1* | PHR1 F | TGAGGAAACTGCAACCCTGA |
|  |  | PHR1 R | ATTCTTCCACACACGTCAGC |
| *CPAR2_109660* | *PHR2* | PHR2 F | CTGATGTTTGGTCTGGTGGT |
|  |  | PHR2 R | AGTTGGGCATGAAGTAACGC |
| *CPAR2_201570* | *ACT1* | Actin F | CGAACGTGGTTACGGTTTCT |
|  |  | Actin R | TGACCATCTGGCAATTCGTA |
